# Small molecule T63 suppresses osteoporosis by modulating osteoblast differentiation via BMP and WNT signaling pathways

**DOI:** 10.1038/s41598-017-10929-3

**Published:** 2017-09-04

**Authors:** Xiao-li Zhao, Jin-jing Chen, Guo-ning Zhang, Yu-cheng Wang, Shu-yi Si, Lin-Feng Chen, Zhen Wang

**Affiliations:** 1 Institute of Medicinal Biotechnology, Chinese Academy of Medical Sciences and Peking Union Medical College, Beijing, 100050 China; 2 Department of Biochemistry, College of Medicine, University of Illinois at Urbana-Champaign, Urbana, IL 61801 USA

## Abstract

Osteoporosis results from the imbalance between bone resorption and bone formation, and restoring the normal balance of bone remodeling is highly desirable for identification of better treatment. In this study, using a cell-based high-throughput screening model representing Runt-related transcription factor 2 (RUNX2) transcriptional activity, we identified a novel small-molecular-weight compound, T63, as an efficient up-regulator of osteogenesis. T63 increased the alkaline phosphatase (ALPL) activity and mineralization as well as gene expression of *Alpl* and other osteogenic marker genes in mouse osteoblasts and mesenchymal stem cell-like cells. Upon induction of osteoblast differentiation, T63 inhibited adipogenic differentiation in the pluripotent mesenchymal cells. Consistently, T63 up-regulated RUNX2 mRNA and protein levels, and knockdown of RUNX2 reduced the osteogenic role of T63. Mechanistically, T63 activated both BMPs and WNT/β-catenin signaling pathways. Inhibition of either signaling pathway with specific inhibitor suppressed T63-induced RUNX2 expression and the osteogenic phenotypes. Moreover, T63 markedly protected against bone mass loss in the ovariectomized and dexamethasone treated rat osteoporosis model. Collectively, our data demonstrate that T63 could be a promising drug candidate and deserves further development for potential therapeutics in osteoporosis.

## Introduction

Osteoporosis is caused by the imbalance between osteoclastic bone resorption and osteoblastic bone formation, and has become one of the major global health concerns during aging development^1^. Either reducing bone resorption or increasing bone formation becomes suitable strategy for treatment of osteoporosis, aiming to restore the normal balance of bone remodeling. Currently, the most used clinical anti-osteoporosis drugs are anti-resorptive agents such as bisphosphonates, denosumab etc.^2^. In addition, inhibiting bone resorption alone is apparently not sufficient, especially for those who already have serious bone mass loss. Thus, developing new approaches that may stimulate osteoblast differentiation is highly desirable.

Osteoblast differentiation is regulated by a variety of factors, including transcriptional factors and signaling pathways, which result in the maturation and mineralization of bone. Runt-related transcription factor 2 (RUNX2) is one of the most important transcription factors in the process of osteogenesis, which triggers the differentiation of mesenchymal stem cells (MSCs) to osteoblasts and functions as a master regulator in osteoblast differentiation at an early stage^3–5^.

RUNX2 binds to a conserved nucleotide sequence (R/TACCRCA), which is named as the osteoblast specific element 2 (OSE2) in osteoblasts, and regulates the transcription of numerous genes, including *alkaline phosphatase* (*Alpl*), bone matrix protein encoding genes *secreted phosphoprotein 1* (*Spp1*, also named as *osteopontin*) and *bone gamma-carboxyglutamate protein* (*Bglap*, also named as *osteocalcin*)^6, 7^, all of which act to induce osteoblastic mineralization^7, 8^. Furthermore, RUNX2 is considered as a pivotal mediator of a variety of signal pathways, including bone morphogenetic proteins (BMPs) and WNT/β-catenin signaling pathways, which are involved in the regulation of osteoblast differentiation^9–13^. Several compounds such as epicatechin gallate, fisetin and resveratrol have been reported to potentiate osteogenesis through activation of RUNX2 transcriptional activity^14–16^. These studies indicate that small molecules up-regulating the RUNX2 transcriptional activity might have therapeutic potentials to treat osteoporosis.

In this study, using a cell-based high-throughput screening monitoring RUNX2 transcription activity, we identified small molecule T63 as a potent agent with osteogenic activity. T63 stimulated osteoblast differentiation by up-regulating the activity of RUNX2. Mechanistic study revealed that T63 potentiated the osteogenic differentiation through activating both BMPs and WNT/β-catenin signaling pathways, leading to the increased RUNX2 expression and its osteogenic activity.

## Results

### A cell-based screening model reflecting RUNX2 transcriptional activity identifies T63 as an up-regulator of RUNX2

**Figure 1:**
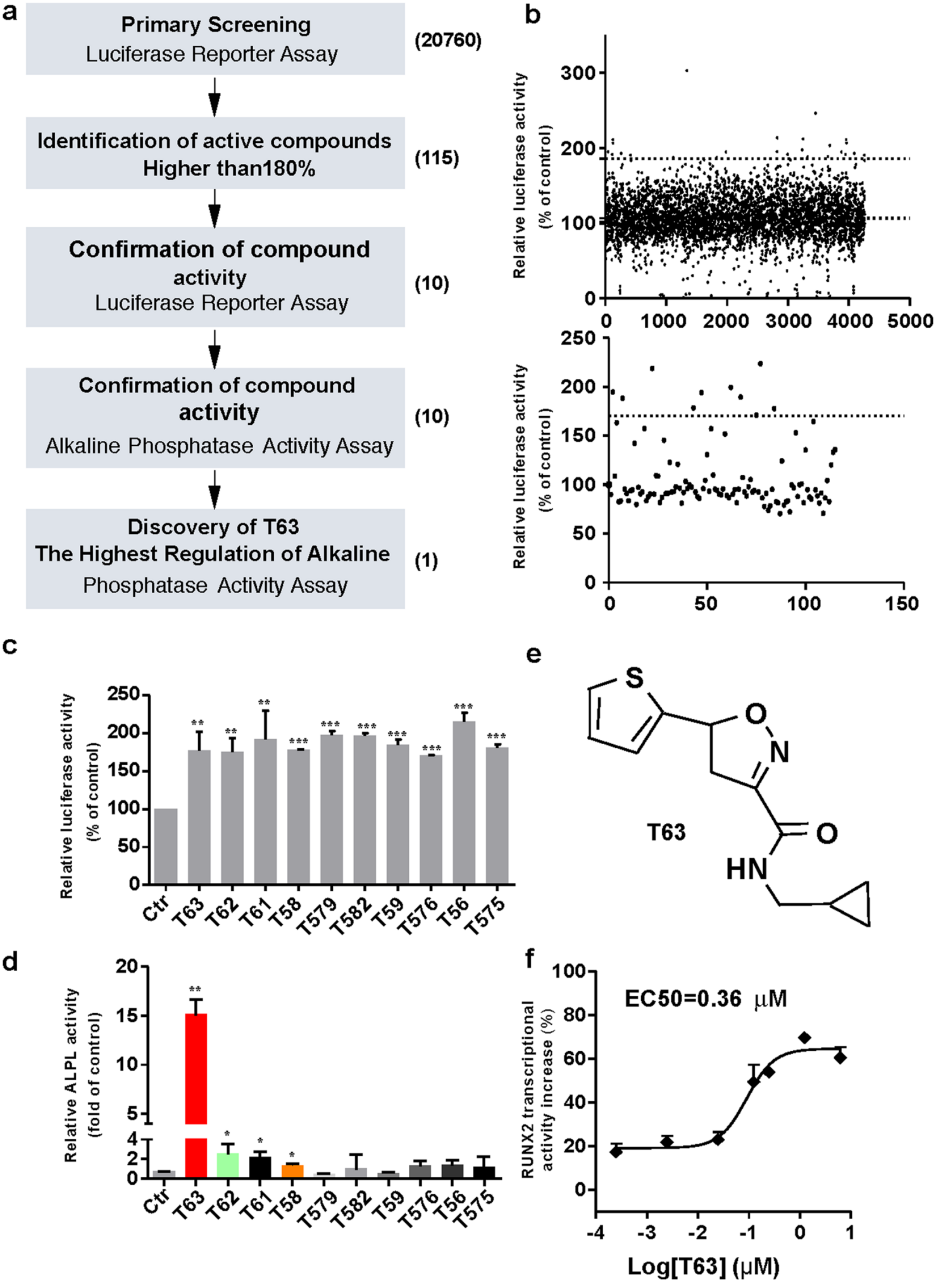
Identification of T63 by high-throughput screening. (**a**) Flow chart of the screening procedure. (**b**) Upper panel: Scatter plot of the first round luciferase-dependent screening in MC3T3-E1-OSE cells. Lower panel: 115 compounds selected for the second round screening. (**c** and **d**) RUNX2 transcriptional activities and ALPL activity of the ten hits (n = 3). *p < 0.05, **p < 0.01, **p < 0.001 versus control. (**e**) Structure of compound T63. (**f**) EC50 curve and value of T63. MC3T3-E1-OSE cells were treated with different concentrations of T63 for 48 h and luciferase activity was measured. EC50 curve and value was performed by Graphpad prism 5 (n = 3).

In order to identify small molecules that could potentially stimulate osteoblast differentiation, we designed a high-throughput screening system that allowed us to measure the transcriptional activity of RUNX2, based on the master regulatory role of the factor in osteogenesis^7, 9^. We generated a mouse preosteoblastic MC3T3-E1 cell line stably expressing pGL4.17-6OSE2-luc, which is responsive to RUNX2 transcriptional activity as described in *Materials and Methods*. We validated the high-throughput screening model by calculating Z’ factor, a characteristic parameter for evaluating the reproducibility and quality of overall assays^17^. The mean Z’ value for total plates in our model was 0.61 ± 0.18, indicating the robustness and reliability of the model. The flow chart for screening is depicted in Fig. 1a. Potential hits with the activity of higher than 180% were selected in the first-round screening from a library of 20760 compounds, resulting in production of 23 stock combinations containing 115 individual compounds (Fig. 1b, upper panel). In the second-round screening, ten compounds (5 μg/ml) were identified to be capable of upregulating RUNX2 transcriptional activity by more than 170% (Fig. 1b, lower panel and Fig. 1c).

To identity compounds that may indeed promote osteogenesis with elevated RUNX2 activity, we further measured the ability of these compounds to activate ALPL, a well-established osteogenesis marker as well as a RUNX2 transcriptional target, four from the ten compounds was found to significantly increase the ALPL activity (*p* < 0.05), with compound T63 showing the highest activity (Fig. 1d). Thus, compound T63 was identified to be an effective up-regulator of osteoblast differentiation, worthy of further study. The chemical name of T63 (PubChem CID: 19582717) is N-(cyclopropylmethyl)-5-(2-thienyl)-1,2-oxazole-3-carboxamide, C_12_H_12_N_2_O_2_S, with the structure shown in Fig. 1e. T63 showed dose-dependent effect on RUNX2 transcriptional activity with EC50 value being 0.36 μM (Fig. 1f). So far, there is only limited evidence suggesting the regulatory role of T63 in neurogenesis and neuronal function^18, 19^. Based on our data, we speculate that T63 might promote osteoblast differentiation.

### T63 potentiates the osteoblast differentiation of mouse osteoblasts and mesenchymal stem cell-like fibroblasts cells

**Figure 2:**
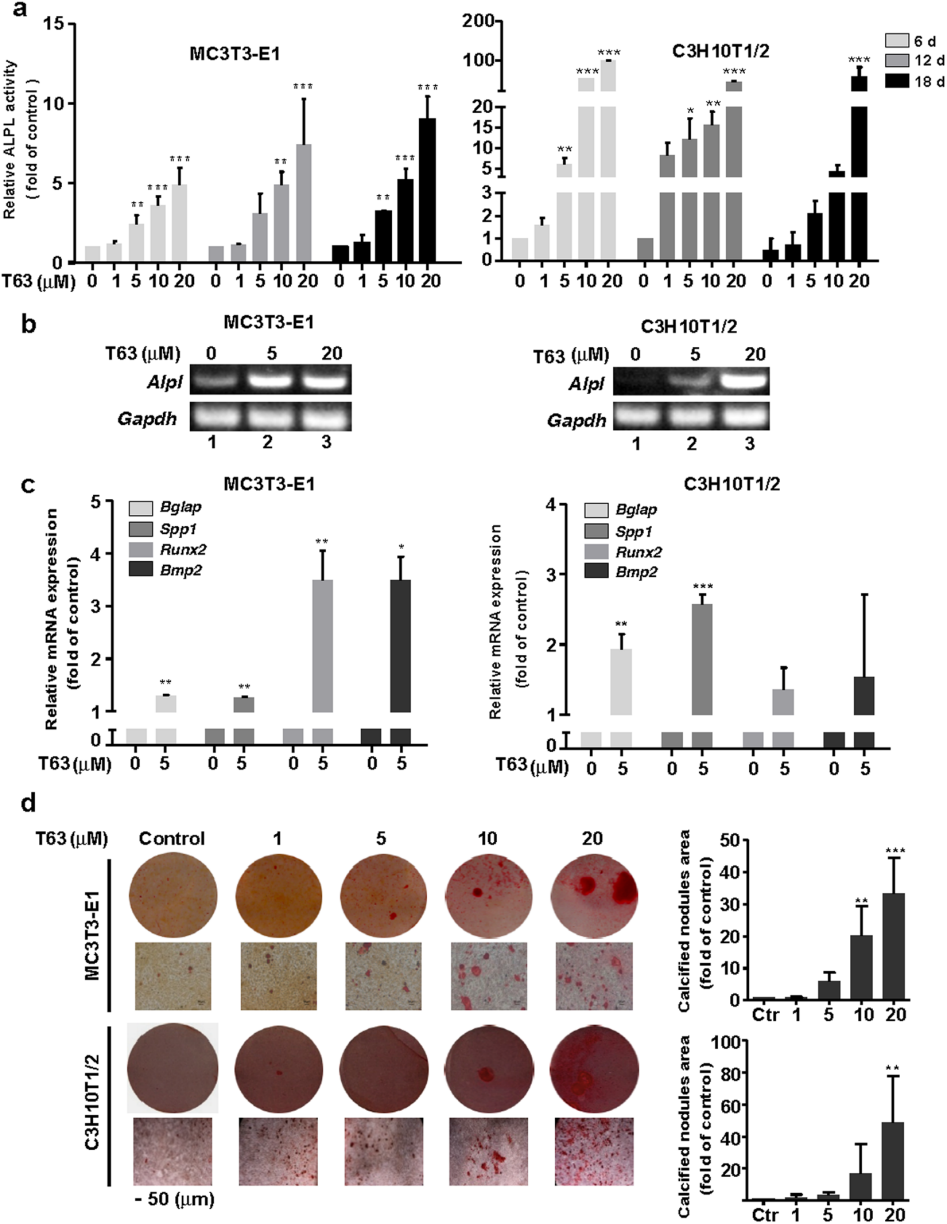
T63 increases osteoblast differentiation in MC3T3-E1 and C3H10T1/2 cells. (**a**) ALPL activity. MC3T3-E1 (left panel) or C3H10T1/2 (right panel) cells were cultured in OS medium, and treated with different concentrations of T63 for the indicated time before subjected to ALPL assay. *p < 0.05, **p < 0.01, ***p < 0.001 versus respective control (n = 4). (**b**) T63 increased *Alpl* mRNA expression. The cells were treated with T63 for 12 days and *Alpl* mRNA was analyzed by semi-quantitative PCR. Full-length gels are shown in Supplementary Fig. S6a. (**c**) Effect of T63 on the mRNA expression of *Bglap*, *Spp1*, *Runx2* and *Bmp2*. The cells were treated with T63 (5 μM) for 12 days before qRT-PCR. *p < 0.05, **p < 0.01, ***p < 0.001 versus control (n = 3). (**d**) Osteoblast mineralization. The cells were cultured in OS medium and treated with T63 for 21 days before Alizarin Red S staining (Scale bar: 50 μm). The quantitation of calcified nodules were shown as means ± SD, **p < 0.01, ***p < 0.001 versus control (n = 3).

We first investigated the role of T63 in osteoblast differentiation in mouse calvarial osteoblasts MC3T3-E1 and mesenchymal stem cell-like fibroblasts C3H10T1/2 cells, both of which can differentiate into osteoblastic cells upon induction in osteogenic supplement (OS) medium. T63 had little cytotoxic effects on both cells after 48 h treatment at 1–40 μM (Supplementary Fig. S1a). To investigate the effects of T63 on osteoblast differentiation, we measured the ability of T63 to activate ALPL activity in MC3T3-E1 and C3H10T1/2 cells cultured in OS medium. T63 significantly increased ALPL activity dose-dependently in MC3T3-E1 cells after treatment for 6, 12 and 18 days, though a time-dependent effect was barely seen (Fig. 2a). Even more significant induction was observed in C3H10T1/2 cells, reaching as high as nearly 100 folds of control after treatment with 20 μM T63 for 6 days (Fig. 2a). *Alpl* mRNA was also markedly and dose-dependently increased after T63 treatment for 12 days (Fig. 2b).

We further investigated the effects of T63 on the expression of other osteogenesis-related genes, including *Bglap* and *Spp1*. T63 (5 μM) markedly increased *Bglap and Spp1* mRNA expressions after treatment for 12 days (Fig. 2c). Notably, the expression of *Runx2* mRNA was significantly induced after 12 days’ treatment (Fig. 2c). Moreover, *Bmp2* mRNA was also up-regulated (Fig. 2c). BMPs is one of the key signals known to be involved in the induction of bone formation as well as the osteogenic effect of other compounds like Salidroside^20–22^. Other BMP family members, including *Bmp4* and *Bmp7*, were similarly induced by T63 (Supplementary Fig. S1b), suggesting that BMP signaling might be involved in T63-induced RUNX2 activation. We next determined the effect of T63 on the cell mineralization by Alizarin Red S staining. T63 significantly increased the mineralization dose-dependently in both cells after treatment for three weeks (Fig. 2d), indicating that T63 stimulates the osteoblast differentiation. Consistently, we also found that T63 has osteogenic effect in human osteoblast cell lines MG63 and hFOB1.19 (Supplementary Fig. S2a–c).

### T63 inhibits the adipogenic differentiation

**Figure 3:**
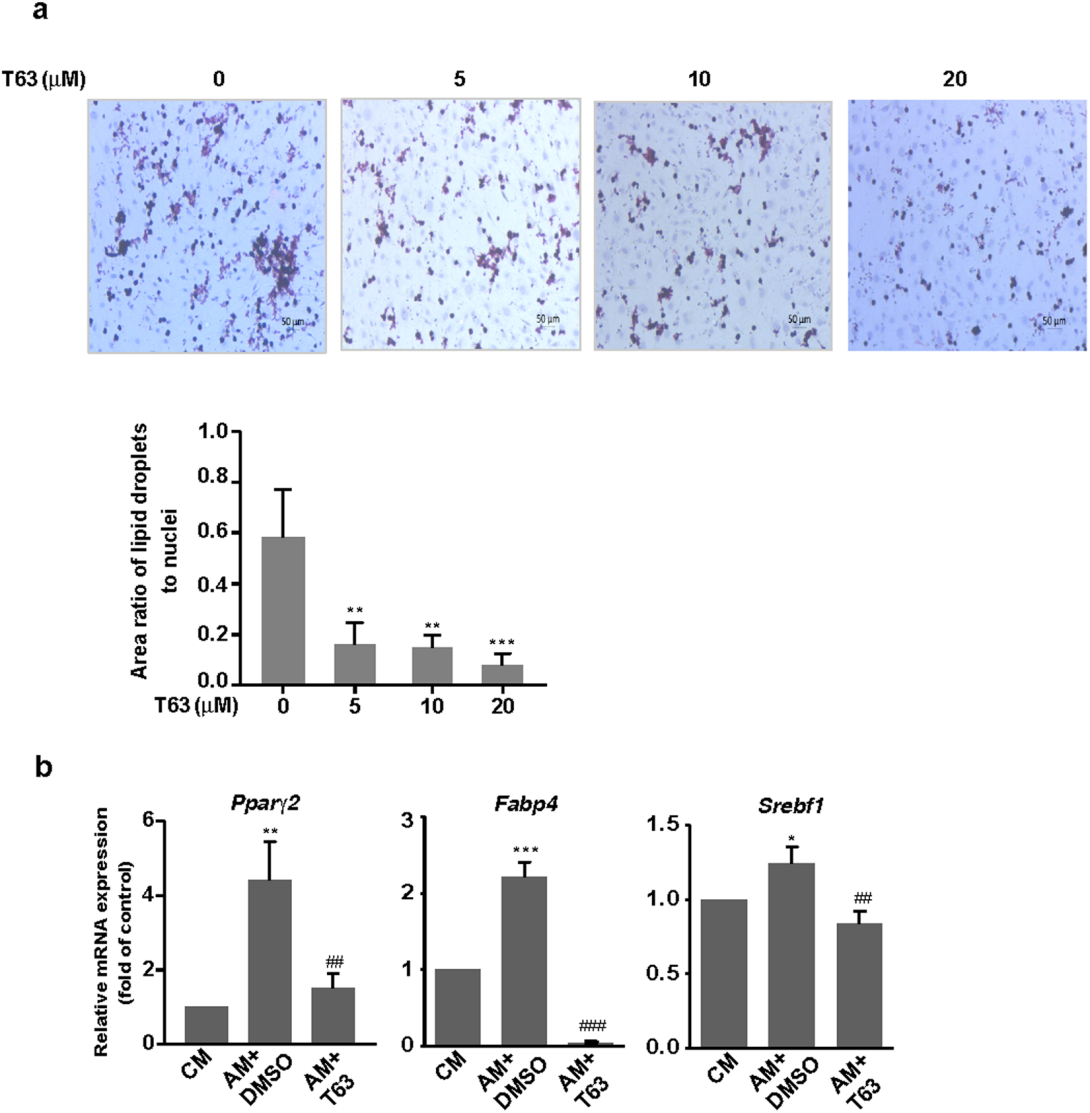
T63 inhibits the adipogenic differentiation of C3H10T1/2 cells. (**a**) Representative images of liqid droplets formation. C3H10T1/2 cells were cultured in the adipogenic medium and treated with T63 for 9 days before Oil Red O staining (Scale bar: 50 μm). The area ratios of lipid droplets to nuclei were quantitated. **p < 0.01, ***p < 0.001 versus control (n = 3). (**b**) Effect of T63 on the mRNA expressions of *Pparγ2*, *Srebf1* and *Fabp4*. C3H10T1/2 cells were cultured in adipogenic medium in the presence or absence of 5 μM T63 for 6 days before qRT-PCR analysis. CM: complete medium; AM: adipogenic medium. *p < 0.01, **p < 0.01, ***p < 0.001 versus CM control, ^##^ *p* < 0.01, ^###^ *p* < 0.001 versus AM control (n = 3).

It has been reported that reciprocal correlation between the differentiation of adipocytes and osteoblasts of MSCs occurs in the bone marrow^5^. Since T63 stimulates the osteoblast differentiation, we then determined whether T63 might affect adipogenic differentiation in C3H10T1/2 cells. The formation of adipocytes was decreased dose-dependently after T63 treatment for 9 days, as measured by Oil Red O staining (Fig. 3a). Consistently, mRNA expressions of adipogenic transcription factors *Pparγ*2 and *Sterol regulatory element binding transcription factor 1* (*Srebf1*) as well as adipogenic marker *Fabp4* (*Fatty Acid Binding Protein 4*) were all significantly suppressed after T63 treatment for 6 days (Fig. 3b). These data suggest that T63 inhibits the adipogenic differentiation while promoting osteoblast differentiation.

### Osteogenic role of T63 is dependent on RUNX2 expression

**Figure 4:**
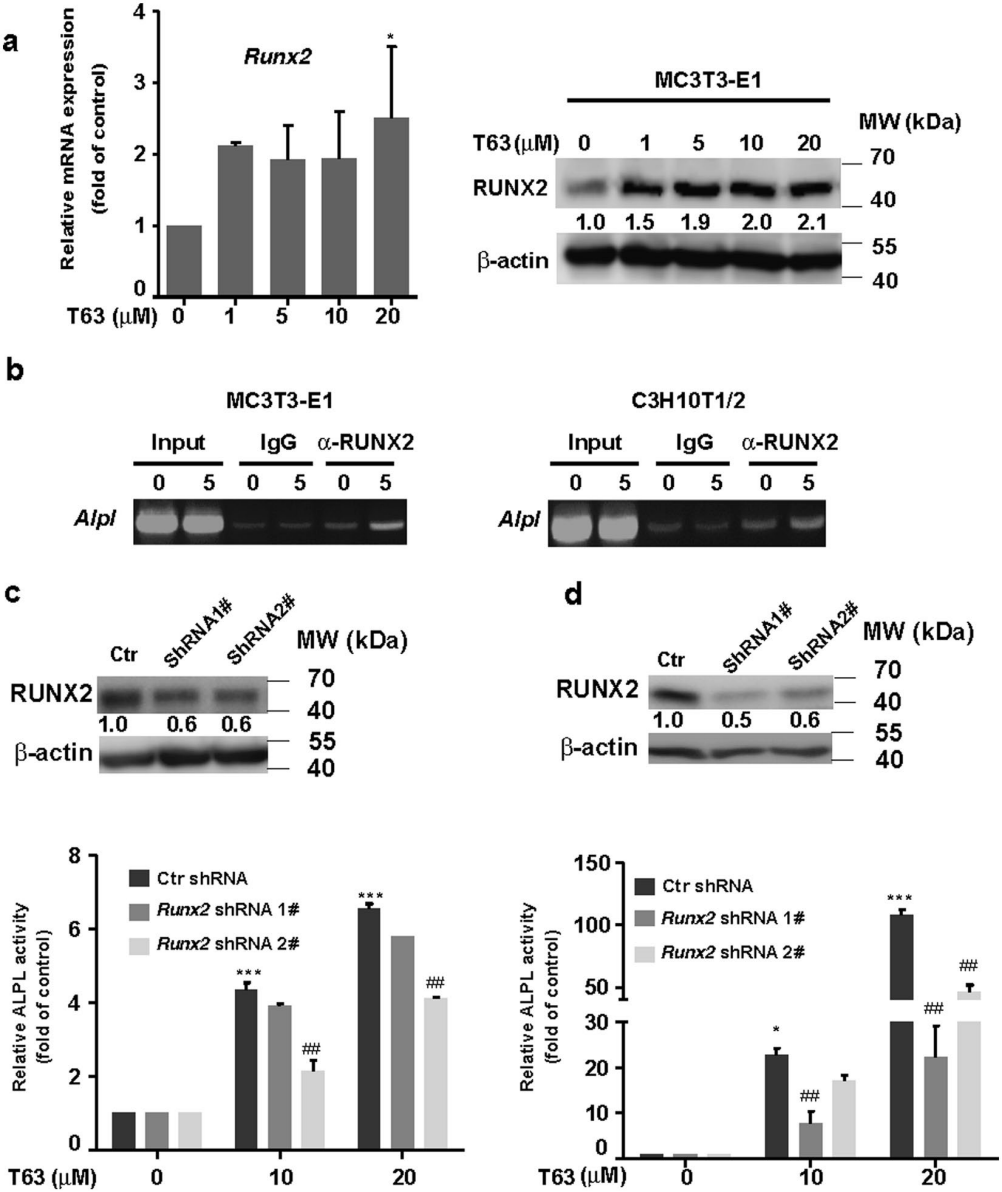
The osteogenic role of T63 is dependent on RUNX2 expression. (**a**) Expression of RUNX2 mRNA and protein. MC3T3-E1 cells were treated with T63 for 48 h, and mRNA (left) and protein level (right) was determined by qRT-PCR and western blot, respectively. *p < 0.05 versus control (n = 3). (**b**) T63 increased the enrichment of RUNX2 on the promoter region of *Alpl* analyzed by ChIP assay. (**c** and **d**) Depletion of Runx2 suppressed T63-induced ALPL activity. MC3T3-E1 (**c**) or C3H10T1/2 (**d**) cells were transfected with Runx2 shRNA for 6 h, followed by T63 treatment for 6 days before subjected to ALPL assay. *p < 0.05, ***p < 0.001 versus drug free control. ^##^ *p* < 0.01 versus respective control shRNA group (n = 3). Knockdown efficiency of Runx2 shRNAs was shown in the upper panels. Full-length blots and gels are shown in Supplementary Figs S5 and S6.

Although *Runx2* mRNA level was increased by T63 after long time treatment (12 days) (Fig. 2c), whether T63 may affect RUNX2 expression at the screening conditions (48 h) remains unknown. We then assessed RUNX2 expression in MC3T3-E1 cells after treatment with T63 of various doses for 48 h, and found that both mRNA and protein level of RUNX2 were increased (Fig. 4a), indicating the increased RUNX2 transcriptional activity stemmed from its expression changes. We also tested the effect of T63 on the expression of *Osterix (Osx)*, another osteoblast-specific transcriptional factor^23^, and found little changes of *Osx* upon T63 exposure (data not shown).

We next investigated whether the osteogenic role of T63 was dependent on RUNX2 expression. Since *Alpl* is a well-characterized target gene of RUNX2 with specific RUNX2 binding site on its promoter^24^, we analyzed the enrichment change of RUNX2 on the promoter region of *Alpl* by ChIP assay. As seen in Fig. 4b, apparent enrichment of RUNX2 protein was observed upon T63 exposure for 6 days in both cells, indicating T63 directly affects the binding of RUNX2 to *Alpl* promoter and thus regulates the activity of ALPL. Accordingly, knockdown of RUNX2 by shRNAs reduced RUNX2 protein expression as well as T63-induced ALPL activity in both cells (Fig. 4c and d).

### BMP and canonical WNT/β-catenin pathways are involved in the regulation of T63-induced RUNX2 expression and osteoblast differentiation

**Figure 5:**
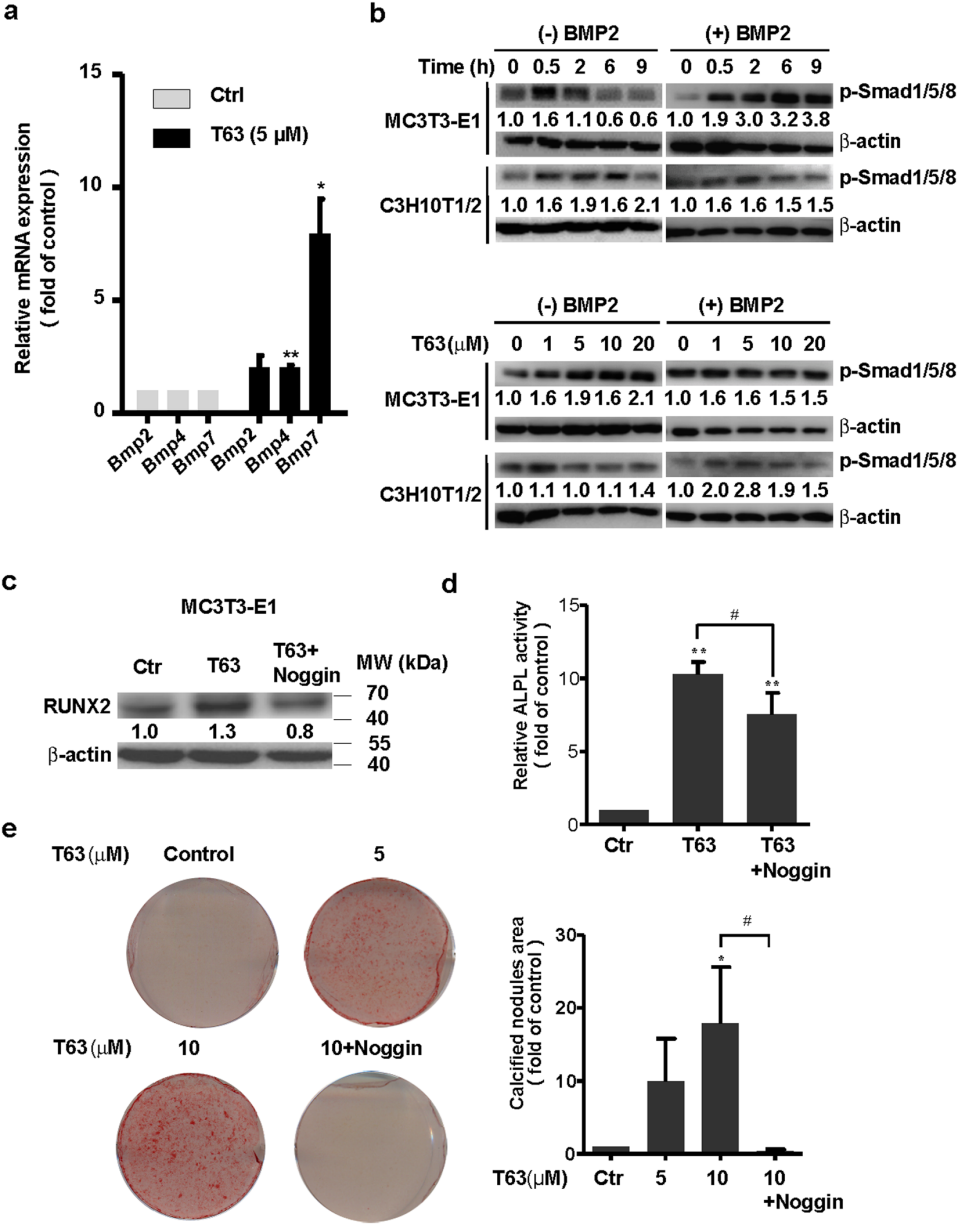
T63 activates BMPs/Smad1/5/8 pathway. (**a**) Expression of *Bmp2*, *Bmp4* and *Bmp7* genes. MC3T3-E1 cells were treated with T63 (5 μM) for 48 h before qRT-PCR analysis (n = 3). *p < 0.05, **p < 0.01 versus control. (**b**) Activation of p-Smad1/5/8 in both dose- and time-dependent manner in the presence or absence of BMP2. The cells were treated with T63 (5 μM) as indicated (upper panels), or with the indicated concentration for 0.5 h (lower panels) in the presence or absence of BMP2 (50 ng/ml), followed by western blot analysis. Relative optical density for each band was quantified, normalized and labeled under each lane. (**c**) Noggin decreased RUNX2 protein level. MC3T3-E1 cells were treated with T63 (5 μM) in the presence or absence of Noggin (200 ng/ml) for 48 h, and subjected to western blot analysis (n = 3). (**d**) Noggin decreased T63-induced ALPL activity. MC3T3-E1 cells were treated with Noggin (200 ng/ml) in the presence of T63 (10 μM) for 6 days and the ALPL activity was measured. **p < 0.01 versus control, ^#^ *p* < 0.05 between both groups (n = 3). (**e**) Noggin decreased T63-induced mineralization. MC3T3-E1 cells were treated with the indicated compounds for 18 days before subjected to Alizarin Red S staining, and the quantification of calcified nodules were plotted as means ± SD (n = 3). *p < 0.05 versus control, ^#^ *p* < 0.05 between both groups. Full-length blots are shown in Supplementary Fig. S5.

We next sought to determine how T63 might regulate RUNX2 expression. As RUNX2 is a crucial mediator of signaling pathways including BMP and WNT/β-catenin signaling pathways, both of which are actively involved in the regulation of osteoblast differentiation^9–13^, we hypothesized that T63 might target these signaling pathways to modulate RUNX2 expression. We first checked BMP signaling pathway. Treatment with T63 for 48 h greatly increased mRNA levels of *Bmp2, Bmp4* and *Bmp7* (Fig. 5a). Further analysis revealed that phosphorylated Smad1/5/8 was increased by T63 in either dose- and time-dependent manner with or without BMP2 stimulation (Fig. 5b), confirming that T63 activated BMP signaling.

We further determined whether BMP signaling was involved in T63-induced RUNX2 expression and osteoblast differentiation. In Fig. 5c, Noggin, a specific inhibitor for BMPs signaling, markedly suppressed T63-induced RUNX2 expression. Consistently, Noggin also inhibited T63-induced ALPL activity as well as the mineralization of the cells (Fig. 5d and e).

**Figure 6:**
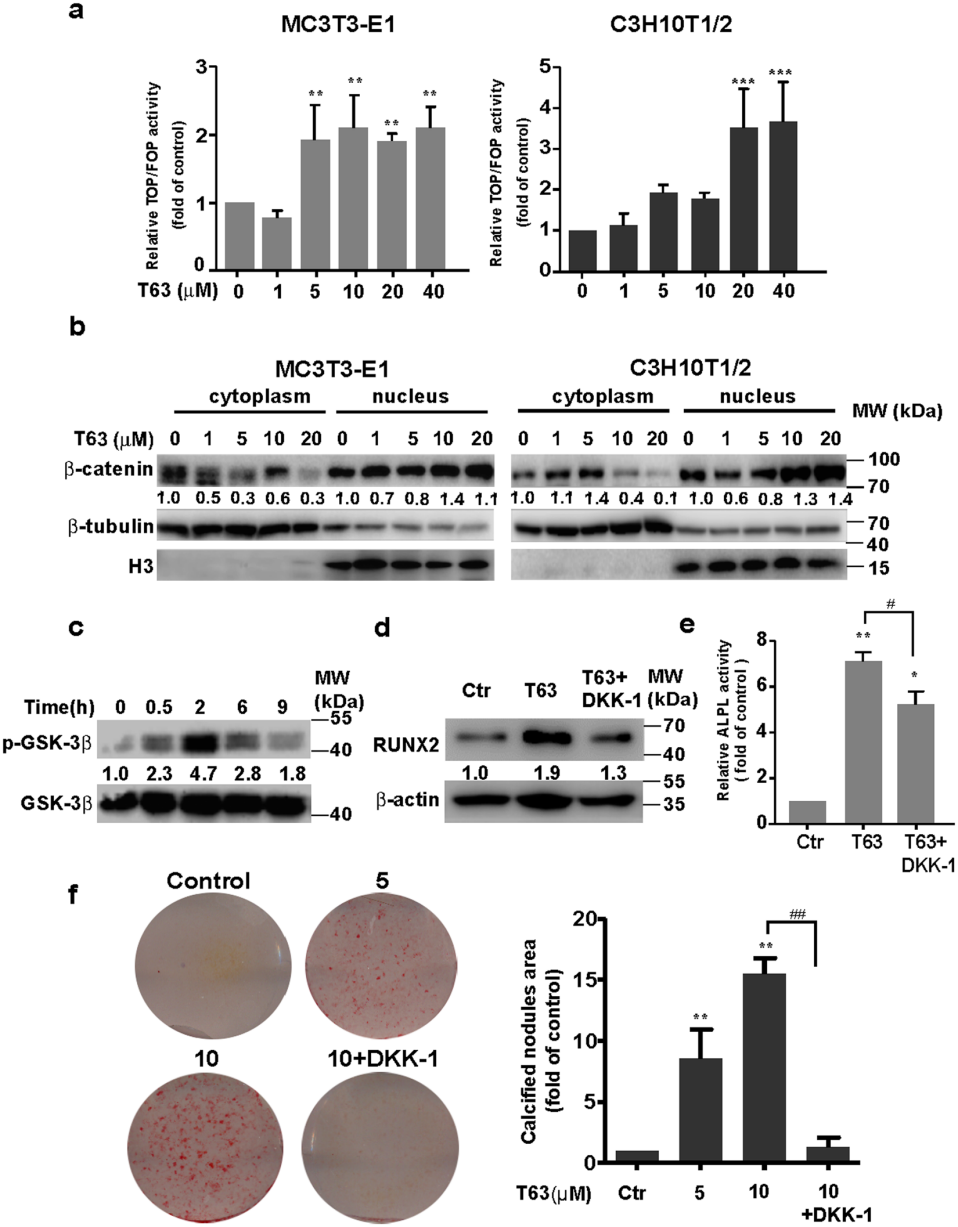
T63 activates canonical WNT/β-catenin pathway. (**a**) T63 increased TCF/LEF reporter activity at 48 h. **p < 0.01, ***p < 0.001 versus respective control (n = 3). (**b**) The levels of β-catenin expression in the nucleus and cytoplasm after treatment with T63 for 48 h. (**c**) MC3T3-E1 cells were treated with T63 (5 µM) as indicated, and the levels of phosphorylated and total GSK-3β proteins were measured. (**d**) DKK-1 decreased RUNX2 protein level. MC3T3-E1 cells were treated with T63 (5 μM) in the presence or absence of DKK-1 (200 ng/ml) for 48 h before western blot analysis. (**e**) DKK-1 decreased ALPL activity. MC3T3-E1 cells were co-treated with T63 (10 μM) and DKK-1 (200 ng/ml) for 6 days in OS medium and ALPL activity was measured. *p < 0.05, **p < 0.01 versus control, ^#^ *p* < 0.05 between both groups (n = 3). (**f**) DKK-1 impaired the mineralization. MC3T3-E1 cells were cultured in OS medium and treated as indicated for 21 days, and the quantification of calcified nodules were plotted as means ± SD (n = 3). **p < 0.01 versus control, ^##^ *p* < 0.01 between both groups. Full-length blots are shown in Supplementary Fig. S5.

We next examined the possible role of WNT/β-catenin signaling pathway. T63 significantly increased TCF/LEF reporter activity, manifesting the enhanced β-catenin transcriptional activity in a dose-dependent manner (Fig. 6a). Moreover, the levels of β-catenin decreased in the cytoplasm but increased in the nucleus upon T63 treatment (Fig. 6b), while total β-catenin level remained unchanged (Supplementary Fig. S3a), indicating the T63 stimulates the nuclear translocation and activation of β-catenin. Further supporting this, the phosphorylation of GSK-3β at Ser9, an upstream regulator of β-catenin, was rapidly up-regulated in MC3T3-E1 cells upon T63 treatment (Fig. 6c). To determine the potential role of WNT pathway in RUNX2 activation and the osteogenic effect, we treated cells with WNT pathway inhibitor DKK-1, and found that DKK1 compromised T63-induced RUNX2 expression, ALPL activity and cell mineralization as well in MC3T3-E1 cells (Fig. 6d,e and f).

As both pathways are reported to have crosstalk regulation during osteogenesis^25–27^, we also found inhibition of BMP signaling by Noggin decreased T63-induced TCF/LEF reporter activity (Supplementary Fig. S3b), possibly indicating the upstream regulatory role of BMP signaling. Collectively, these data demonstrate that BMP and WNT/β-catenin signaling pathways are actively involved in T63-induced RUNX2 activation and osteoblast differentiation.

### T63 attenuates bone mass loss in OVX-D rat osteoporosis model

**Figure 7:**
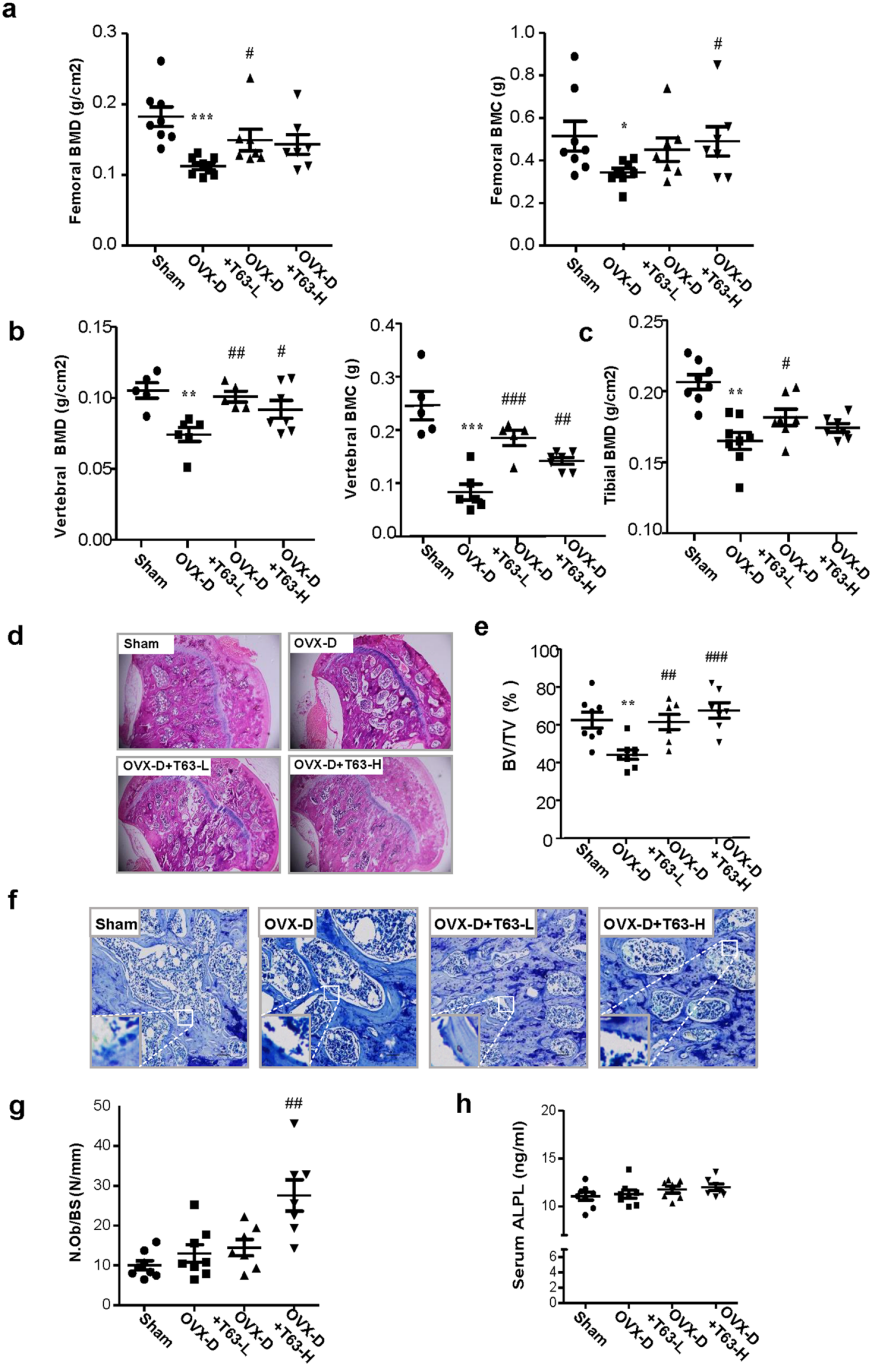
T63 attenuates bone mass loss in OVX-D rat osteoporosis model. (**a**,**b**) BMD and BMC of the right femur and lumbar vertebrae. *p < 0.05, **p < 0.01, ***p < 0.001 versus Sham group, ^#^ *p* < 0.05, ^##^ *p* < 0.01, ^###^ *p* < 0.001 versus OVX-D group. (**c**) BMD of the right tibia. **p < 0.01 versus respective Sham group, ^#^ *p* < 0.05 versus OVX-D group. (**D**) Femoral H&E staining (×5 magnification). (**e**) Bone histomorphometric analysis of bone volume fraction (BV/TV%). **p < 0.01 versus Sham group. ^##^ *p* < 0.01, ^###^ *p* < 0.001 versus OVX-D group. (**f**) Femoral toluidine blue staining (Scale bar: 100 μm). (**g**) The numbers of osteoblasts (N.Ob) per millimeter of trabecular bone surface (BS) were counted. ^##^ *p* < 0.01 versus OVX-D group. (**h**) Serum ALPL level.

We next determined the potential therapeutic effects of T63 for osteoporosis *in vivo*. In the OVX-D-induced osteoporosis rat model, two groups of rats were administrated with two different doses of T63 daily (5 mg/kg and 20 mg/kg, designated as T63-L and T63-H, respectively) for three months. The femurs and lumbar vertebrae in the sacrificed rats were collected for analysis. As expected, bone mineral density (BMD) and bone mineral content (BMC) in femurs and lumbar vertebrae were significantly lowered in the OVX-D model group, while markedly increased in T63-L and T63-H groups compared with the OVX-D control group (Fig. 7a and b), indicating that T63 significantly increased bone formation, although dose-dependent effect could be hardly seen. Meanwhile, T63 had little effect on the body weight of the animals, suggesting the low toxicity of T63 (Supplementary Fig. S4a). Tibial BMD had similar tendency as that of femurs and lumbar vertebrae (Fig. 7c). Moreover, treatment with T63 significantly improved the altered femur structure as observed in OVX-D group (Fig. 7d). Consistently, the femoral BV/TV% was significantly increased in both T63 groups, as compared with that in the OVX-D group (Fig. 7e). We further checked effects of T63 on osteoblasts amounts of trabecular bone surface of the femurs by histomorphometric analysis, and found the osteoblasts number was significantly increased in T63-H group (*p* < 0.01) (Fig. 7f and g), while T63 slightly increased serum ALPL level (Fig. 7h). We also checked whether T63 affects osteoclasts amounts in the trabecular bone surface. As shown in Supplementary Fig. S4b and c, T63 reduced TRAP-positive staining area in the femurs and serum NTX-1 level, a marker reflecting osteoclast function, as compared with OVX-D group, indicating the compound may regulate bone resorption as well.

## Discussion

In this study, we established and validated a cell-based high-throughput screening model to identify small molecules that would enhance RUNX2 activity and stimulate osteoblast differentiation, and identified a novel small-molecular-weight compound T63 as an effective up-regulator of RUNX2 activity (Fig. 1). T63 effectively stimulates osteoblast differentiation, as evidenced by increased ALPL activity and cell mineralization along with enhanced expression of *Alpl* and other osteogenic marker genes. Moreover, our *in vivo* data confirmed the protective role of T63 against bone loss observed in the OVX-D rat osteoporosis model.

T63-induced RUNX2 activation occurs at the transcription level, since *Runx2* mRNA was increased by T63 (Figs 2c and 4a). Also, the osteogenic role of T63 was dependent on RUNX2 expression and activity, as T63 stimulated the binding of RUNX2 to the promoter of *Alpl* and *RUNX2* knockdown markedly reduced ALPL activity (Fig. 4). Meanwhile, the osteogenic effects of T63 doesn’t seem to result from the increased cell number, since T63 had little effect on the cellular viability in osteoblasts (Supplementary Fig. S1a).

RUNX2 and PPARγ are two pivotal transcription factors that are involved in the regulation of osteogenic and adipogenic lineage differentiation of MSCs^28, 29^. Some phytoestrogens such as resveratrol are reported to reciprocally regulate the osteoblast differentiation and adipogenesis^16^. We also found that T63, upon the induction of osteogenic differentiation, could inhibit the adipogenic differentiation in C3H10T1/2 cells (Fig. 3). Since RUNX2 transcriptional activity could be negatively regulated by PPARγ2^30^, whether T63 might affect RUNX2 transcription indirectly through inhibiting PPARγ2 expression remains unknown and merits further investigation.

The regulation of RUNX2 activity by T63 appears to be indirect via BMPs/Smads and WNT/β-catenin signalings, both of which converge to regulate RUNX2 expression^10, 11^. BMPs, belonging to the members of TGF-β superfamily, have essential roles in osteoblast differentiation^20, 21, 31^. Among many BMPs family members, BMP2 is a pivotal signal, and upon activation, recruits phosphorylated Smad1/5/8 to form a complex with Smad4, which then translocates into the nucleus to activate the transcription of *Runx2* ^31, 32^. Also, canonical WNT/β-catenin signaling has well-established roles in regulating osteogenesis by increasing bone mass in animal models^33^. When activated, WNTs evoke the cascade of a serial of downstream events, which results in the translocation of β-catenin into the nucleus to initiate the transcription of a number of target genes, including *Runx2* ^10, 34^. Here, we found that both BMP/Smads and canonical WNT signaling pathways were actively involved in the regulation of RUNX2 activity. Moreover, our result suggested the BMP signaling might function at the upstream of Wnt/β-catenin signaling pathway (Supplementary Fig. S3b). Supportively, BMP is found to promote WNT/β-catenin signaling with multiple mechanisms during osteogenesis regulation^25–27^. However, based on the complexity for crosstalk regulation of these signaling pathways^26^, the detailed regulation regarding how T63 may affect these pathways remains to be determined. Also, given the fact that WNT signaling pathway not only increases RUNX2 activity but also decreases PPARγ2 activity^4, 35^, we speculate that the inhibition of PPARγ*2* expression by T63 might be mediated by WNT signaling.

In this study, we used an osteoporosis animal model by combining ovariectomy with dexamethasone treatment (OVX-D) in rats. This model, mimicking human postmenopausal osteoporosis, can induce more rapid and severe osteoporosis phenotypes compared with ovariectomized model^36^. Meanwhile, despite enhanced osteoclastogenesis, the combined treatment may suppress osteogenesis given that dexamethasone can directly induce osteoblast cell death through activating glucocorticoid receptors^37^. In addition, the combination treatment down-regulates RUNX2 expression^38^. Thus, this model is suitable for testing the effect of T63 *in vivo*.

We found that T63 significantly enhanced bone mass and restored the altered bone structure in the OVX-D model (Fig. 7). While only marginal increase of serum ALPL activity was seen upon T63 exposure, the compound at high dose indeed increased the osteoblasts number on the surface of trabecular bone (Fig. 7f and g). Interestingly, T63 is also found to reduce the increased osteoclasts number and function from the model (Supplementary Fig. S4). Based on the fact that RUNX2 is capable of inhibiting the osteoclastic differentiation by promoting the expression of osteoprotegerin (OPG), a potent inhibitor of osteoclast differentiation^39^, and that T63 was consistently found to increase the OPG/RANKL ratio in human osteosarcoma cells^40^, it is thus possible the anti-osteoclastic effect of T63 *in vivo* might be mediated by the elevated RUNX2 activity as well. Supportively, our *in vitro* study using a co-culture system combining MC3T3-E1 cells with RAW264.7 cells showed that T63 reduced *Rankl* mRNA expression in the osteoblasts, while inhibiting the osteoclastic differentiation of RAW264.7 cells (data not shown). Collectively, we speculate that T63 may regulate bone metabolism by restoring the imbalance between osteoblasts and osteoclasts *in vivo*.

Taken together, a small molecule T63 is identified as a RUNX2 transcriptional activator, which potentiates the osteogenic differentiation and bone formation. The osteogenic effect of T63 activates is mediated by BMPs and WNT/β-catenin signaling pathways. With its small molecular weight and easy synthesis, T63 merits further development for the potential therapeutic purpose in the treatment of osteoporosis.

## Methods

### Reagents and cell lines

The compound library used for HTS was purchased from Enamine Ltd. (Kiev, Ukraine). T63 was obtained from J&K Scientific Ltd. (Beijing, China). Human osteoblast-like cell line MG63 and MC3T3-E1 mouse calvarial preosteoblasts were obtained as described^41^. C3H10T1/2 mouse pluripotent mesenchymal stem cell-like fibroblasts were obtained from the Bank of Type Culture Collection of Chinese Academy of Sciences (Shanghai, China). Human fetal osteoblastic cell line hFOB1.19 cells were from Shanghai Zhong Qiao Xin Zhou Biotechnology Co.,Ltd (Shanghai, China). All antibodies were purchased from Cell Signal Technology (Beverly, MA, USA) except for β-actin from Sigma-Aldrich. Horseradish peroxidase-linked secondary antibodies were purchased from Santa Cruz Biotechnology (CA, USA). TRIZOL reagent was purchased from Life Technologies (Carlsbad, CA, USA). Recombinant human BMP2 was obtained from Sino Biological Inc. (Beijing, China). All other reagents are from Sigma-Aldrich (St. Louis, MO, USA).

### Cell culture

MC3T3-E1, C3H10T1/2 cells, MG63 and hFOB1.19 were cultured in α-MEM or MEM or DMEM or DMEM/F-12 (0.3 mg/ml G418) medium, respectively (Hyclone, Logan, Utah, USA), supplemented with 10% FBS (Life Technologies), 100 units/ml penicillin and 100 mg/L streptomycin (Amresco, Solon, OH, USA). For osteoblast differentiation induction, the cells were cultured in OS medium, respectively. For MC3T3-E1 cells, the OS medium was complete medium containing 5 mM β-glycerophosphate (β-GP) and 25 μg/ml ascorbic acid (Vc), for C3H10T1/2 cells, the OS medium was complete medium containing 5 mM β-GP, 25 μg/ml Vc and 10 nM dexamethasone (Dex), and for MG63 cells, the OS medium was complete medium containing 10 mM β-GP, 50 μg/ml Vc and 10 nM Dex and for hFOB1.19 cells, the OS medium was complete medium containing 10 mM β-GP, 50 μg/ml Vc and 100 nM Dex. The medium was changed every three days.

### Establishment of high-throughput screening model

The MC3T3-E1-OSE cells were seeded in 96-well plates and treated with stock compound combinations (with five compounds in one combination) at the final concentration of 5 μg/ml for each compound. After 48 h, the cells were lysed and the luciferase acvtivity was measured using the Luciferase Assay System (Promega, Madison, WI, USA). The activity of negative control in 0.1% DMSO was defined as the basis of 100% activity, and the relative luminescence units (RLUs) of the tested compounds was calculated by 100% × RLUs _test compound_/RLUs _negative control_. The stock compound combinations with potential activity of higher than induction ≥ 3 S.D. from average (i. e. 180%) were considered as primarily positive and individual compounds in each combination were selected for re-confirmation test in triplicate in a second round test. EC50 values were calculated by Graphpad prism 5.0 software.

### ALPL assay

ALPL activity was measured as described previously^42^. Specifically, the cells were seeded in 6-well plate and treated as indicated. After treatment, the cells were sonicated and the supernatants were collected and incubated with 100 μl pNPP substrate solution containing 1.0 mg/mL pNPP, 1 M diethanolamine buffer and 0.5 mM magnesium chloride (pH = 9.8) for 30 min at 37 °C, then stopped with 3 M NaOH solution and measured at 405 nm using the microplate reader (Bio-Rad). Each experiment was performed in triplicates, and repeated at least three times.

### Alizarin Red S staining

The cells were seeded in 6-well plates at the density of 10^5^ cells per well and treated as indicated. After treatment, the cells were fixed with 70% ethanol for 1 h, and then stained with 40 mM of Alizarin red S solution (pH 4.1–4.3) for 10 min at room temperature. Positive staining area indicating the calcified nodules per field was counted with Image-Pro plus software (Media Cybernetics Inc., MD, USA) and normalized to respective control.

### Oil Red O staining

C3H10T1/2 cells were cultured in adipogenic medium consisting of complete medium with 5 µg/ml insulin, 0.5 mM IBMX and l μM dexamethasone in the presence of T63 at final concentrations of 5–20 μM for 9 days. Differentiated C3H10T1/2 cells were fixed with 70% ethanol for 1 h and rinsed with 60% isopropanol solution, followed by staining with Oil Red O solution for 20 min. Lipid droplets formation were visualized and images were captured by inverted microscopy. Positive staining area demonstrating lipid droplets formation was quantified using Image-Pro plus software, and the area ratio (%) of lipid droplets was normalized to the number of nuclei.

### Western blot analysis

Protein extracts (25–50 μg) were resolved by SDS-polyacrylamide gel electrophoresis, and transferred to PVDF membrane (Millipore Corporation, Billerica, MA). The membranes were blocked with 5% nonfat milk PBS-T buffer at room temperature for 1 h, and then incubated with primary antibodies for 2 h at 1:1,000 dilution, except for β-actin (1:5,000). The membranes were then incubated for 1 h with horseradish peroxidase-linked secondary antibody, and electrochemiluminescence was performed with ChemiImager 5500 imaging system (Alpha Innotech Corporation, San Leandro, CA). Data shown in the results are representative of at least three independent experiments. Optical density of representative blots were determined as described previously^43^.

### RT-PCR assay

RNA was extracted and reversely transcribed using PrimeScript RT Master Mix (TaKaRa, Japan). The gene expression patterns were quantified using SYBR Green-based real-time PCR (CFX96 qRT-PCR, Bio-Rad). Fold changes were calculated using the 2^−ΔΔCt^ method of relative gene quantification and normalized to the house-keeping gene *Gapdh*. Each experiment was performed in triplicates and repeated three times. For semi-quantitative PCR, the amplified products were visualized by gel electrophoresis in 2% agarose and stained with 0.5 µg/ml ethidium bromide. The primers were listed in Table 1.

**Table 1:** The primers.

Primer name	Sequence (5′-3′)	
*Runx2*	F: GAATGCACTACCCAGCCAC	R: TGGCAGGTACGTGTGGTAG
*Alpl*	F: TGACCTTCTCTCCTCCATCC	R: CTTCCTGGGAGTCTCATCCT
*Spp1*	F: TCCAAAGCCAGCCTGGAAC	R: TGACCTCAGAAGATGAACTC
*Bglap*	F: CAATAAGGTAGTGAACAGAC	R: CTTCAAGCCATACTGGTCT
*Bmp2*	F: CTGACCACCTGAACTCCAC	R: CATCTAGGTACAACATGGAG
*Bmp4*	F: GACTTCGAGGCGACACTTCT	R: GCCGGTAAAGATCCCTCATGTA
*Bmp7*	F: GAAAACAGCAGCAGTGACCA	R: GGTGGCGTTCATGTAGGAGT
*Pparγ2*	F: CACCAGTGTGAATTACAGCAAATC	R: ACAGGAGAATCTCCCAGAGTTTC
*Srebf1*	F: GATGTGCGAACTGGACACAG	R: CATAGGGGGCGTCAAACAG
*Fabp4*	F: AAGGTGAAGAGCATCATAACCCT	R: TCACGCCTTTCATAACACATTCC
*Gapdh*	F: CATGGCCTTCCGTGTTCCTA	R: CCTGCTTCACCACCTTCTTGAT

### Gene knockdown

*Runx2* gene was knocked down with shRNAs (origene #TR510502), with sequences as follows: GCAAGAGTTTCACCTTGACCATAACAGTC (1#), CCTAGTTTGTTCTCTG-ATCGCCTCAGTGA (2#). The transfection was performed with Lipofectamine^TM^ LTX (Life technologies) according to the manufacturer’s recommendation.

### ChIP Assay

ChIP assay was performed as described previously^43^. The presence of *Alpl* promoter domain in immunoprecipitated DNA was identified using the primers as described before^24^. Reactions were set for denaturation at 94 °C for 1 min and annealing at 59 °C for 1 min, followed by elongation at 68 °C for 2 min. The amplified products were observed after 35 cycles and visualized by gel electrophoresis in 2% agarose and stained with 0.5 µg/ml ethidium bromide.

### Dual luciferase assay

The cells were seeded in 96-well plates at 1 × 10^4^ cells/well for 20 h. Then the cells were transfected with the 0.1 µg TOPflash or FOPflash (upstate) along with 10 ng pRL-TK reporter vector in triplicate using Lipofectamine^TM^ LTX (Life Technologies). After transfection for 6 h, the cells were treated with T63 and luciferase activity was measured using the dual-luciferase reporter assay system (Promega). Each experiment was performed in triplicates and repeated three times.

### *In vivo* experiment

Rat osteoporosis model was established with minor modification by combining ovariectomy with dexamethasone treatment (abbreviated as OVX-D) as previously reported^36, 38^. Thirty-two 7-month-old female SD rats (290–310 g) (Beijing Vital River Laboratory Animal Technology Co., Ltd, China) were anaesthetized by intraperitoneal injection of pentobarbital sodium (40 mg/kg), and twenty-four rats were bilaterally ovariectomized, while the ovaries of the remaining animals were left intact (Sham operation). One week later, the ovariectomized rats were intramuscular injected with dexamethasone (1 mg/kg) twice a week for four consecutive weeks. All the OVX-D-induced rats were then randomly divided into three groups with eight rats in each group, including control (OVX-D), low-dose (T63-L) and high-dose (T63-H) group, which was given with 0.3% CMC-Na, 5 mg/kg/d of T63 and 20 mg/kg/d of T63, respectively, by intragastrical administration daily for three months. Four rats were housed in each cage under well-controlled temperature (24–26 °C) and allowed free access to forage and water. During the whole procedure, only one rat in T63-L group and one in T63-H group died for unknown reason. After treatment, femur, tibia and lumbar vertebrae (L3-L4) from the animals were collected to determine the bone mineral density (BMD) and bone mineral content (BMC) using dual-energy X-ray absorptiometry (DXA). For bone histological analysis, the femurs were fixed in 4% paraformaldehyde, decalcified in 10% EDTA (pH 7.2), followed by being stained with H&E and toluidine blue to visualize osteoblasts. The numbers of osteoblasts (N.Ob) per millimeter of trabecular bone surface (BS) and Bone volume fraction (BV/TV%) were analyzed by Image Pro Plus 6.0 as described^44^. For histomorphometry analysis, three random fields near epiphysis per sample were counted and averaged for each sample. ALPL and NTX-1 level in the serum of the animals was measured using rat ALPL ELISA kit (Nanjing Sen Bei Jia Biotechnology Co.,LTD, China) and NTX-1 ELISA kit, respectively (Beijing Fangchengbaijin Technology Co., LTD, China). The animal study was approved by the Institutional Animal Care and Use Committee (IACUC) of Institute of Medicinal Biotechnology. We confirm that all methods were performed in accordance with the relevant ethical guidelines and regulations.

### TRAP staining

Femoral sections were deparaffinised and TRAP staining was performed using a commercial acid phosphatase leucocyte kit (Sigma, 386 A).

### Statistical analysis

Data were expressed as means ± SD and analyzed using SPSS 13.0. Statistical significance was determined by One-way analysis of variance (ANOVA) followed by LSD test or Tamhane’s test after homogeneity of variance test. Differences between the groups were identified as statistically significant at three levels: *p* < 0.05, *p* < 0.01, and *p* < 0.001.

## Electronic supplementary material

Supplementary information
