# Supplementary material for: Small molecule T63 suppresses osteoporosis by modulating osteoblast differentiation via BMP and WNT signaling pathways
Source: Sci Rep. 2017 Sep 4;7:10397. doi: 10.1038/s41598-017-10929-3 (PMC5583318; doi:10.1038/s41598-017-10929-3)
Supplement: Supplementary file 1 — Supplementary information [file 41598_2017_10929_MOESM1_ESM.pdf]

Small molecule T63 suppresses osteoporosis by modulating osteoblast differentiation via BMP and WNT signaling pathways

Xiao-li Zhao, Jin-jing Chen, Guo-ning Zhang, Yu-cheng Wang, Shu-yi Si, Lin-Feng Chen*, Zhen Wang*

S1

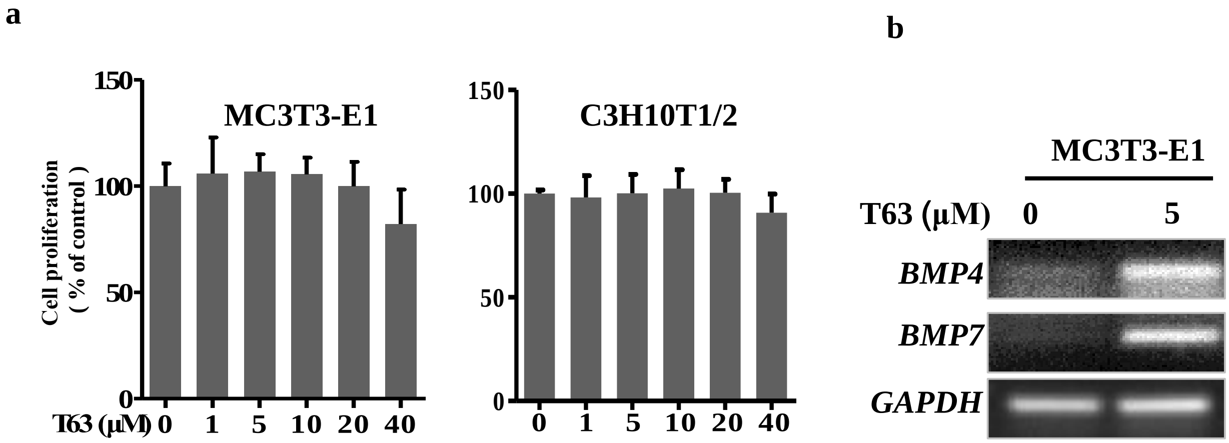

S2

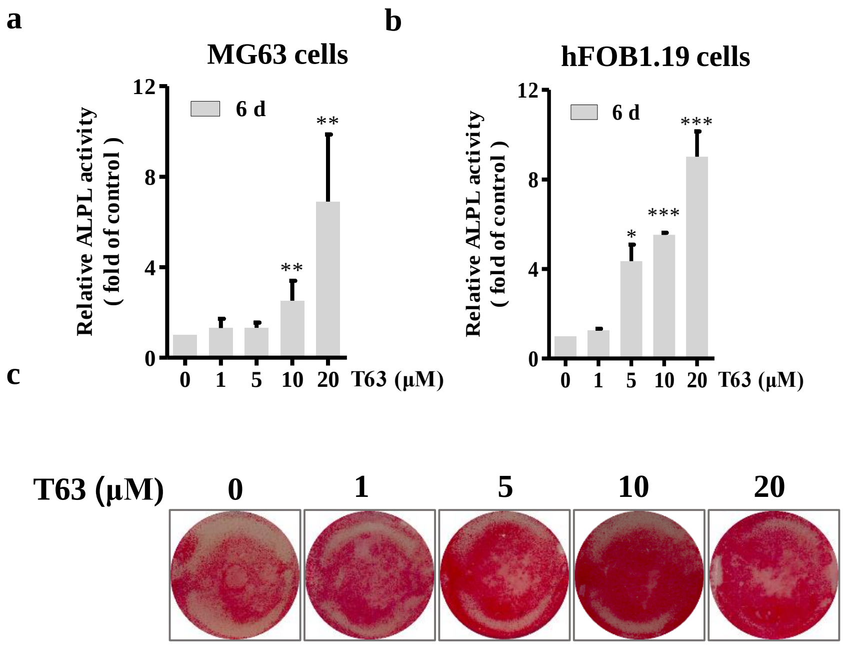

S3

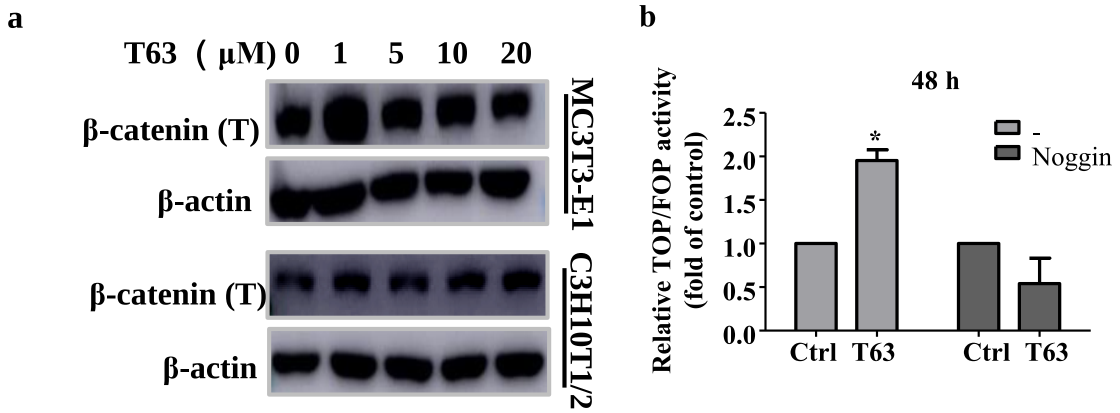
a
T63 (μM) 0 1 5 10 20
β-catenin (T)
β-actin
β-catenin (T)
β-actin
MC3T3-E1
C3H10T1/2
b
48 h
Relative TOP/FOP activity (fold of control)
2.5
2.0
1.5
1.0
0.5
0.0
*
-
Noggin
Ctrl T63 Ctrl T63

S4

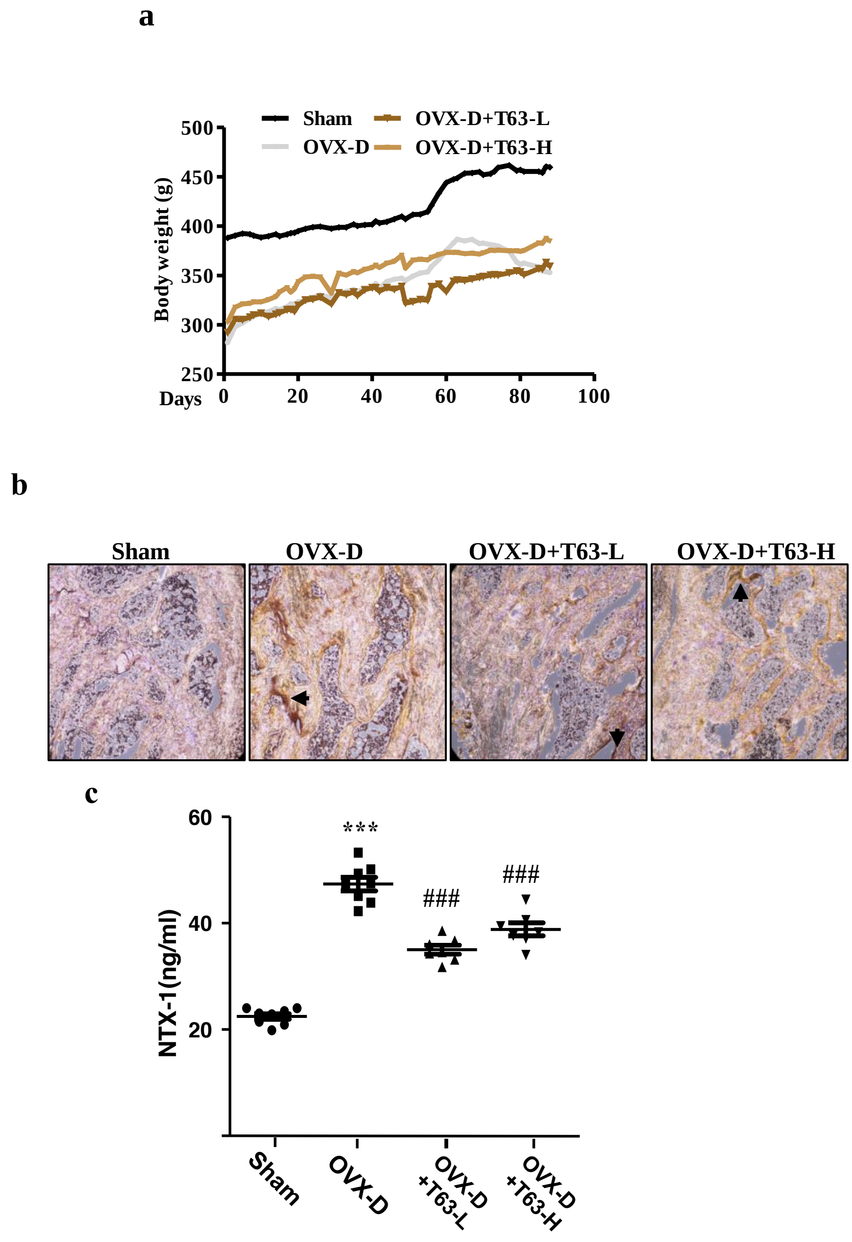
a
Sham
OVX-D
OVX-D+T63-L
OVX-D+T63-H
Body weight (g)
500
450
400
350
300
250
Days 0 20 40 60 80 100
b
Sham
OVX-D
OVX-D+T63-L
OVX-D+T63-H
c
NTX-1(ng/ml)
60
40
20
***
###
###
Sham
OVX-D
OVX-D +T63-L
OVX-D +T63-H

Supplementary Figure S1. Effect of T63 on the proliferation and osteoblastic differentiation of C3H10T1/2 and MC3T3-E1 cells. (a) C3H10T1/2 or MC3T3-E1 cells were seeded in 96-well plate for 24 h, and then treated with different concentrations of T63 for another 48 h. Cell proliferation was determined by MTT assay. (b) Expression of BMP4 and BMP7 genes in T63-treated MC3T3-E1 cells. The cells were treated with 5 μM T63 for 12 days in OS medium and mRNA expressions were determined by semi-quantitative PCR.

Supplementary Figure S2. Effect of T63 on osteoblastic differentiation of human cells. (a)& (b) The levels of ALPL activity. The cells were seeded in 6-well plates (2×10^5 cells /well) for 24 h and treated with different concentrations of T63 in OS medium for 6 d.**p < 0.01,***p < 0.001. (c) Osteoblast mineralization was observed by Alizarin Red S staining in MG63 cells for 18 d.

Supplementary Figure S3. BMP and canonical WNT/β-catenin pathways are involved in the regulation of T63-induced RUNX2 expression and osteoblast differentiation. (a) T63 activates canonical WNT/β-catenin signaling. MC3T3-E1 cells were seeded in 6-well plates (3×10^5 cells /well) for 24 h and treated with different concentrations of T63 for another 48 h. The levels of indicated proteins were determined by western blot. (b) Noggin（200 ng/ml）suppresses T63 increased- TCF/LEF reporter activity at 48 h in MC3T3-E1 cells, *p < 0.05 (n=3).

Supplementary Figure S4. T63 attenuates OVX-D-induced bone loss. (a) Body weight of each rat was measures every one or two days. (b) TRAP staining was performed on femur sections.(c) Serum NTX-1 level.***p < 0.001 versus Sham group. ###p < 0.001 versus OVX-D group.

S5. Uncropped western blots for the blots used Figures

Fig. S5a: full Western blot of Fig. 4a.

Fig. S5b: full Western blot of Fig. 4c.

Fig. S5c: full Western blot of Fig. 4d.

Fig. S5d: full Western blot of Fig. 5b.

Fig. S5e: full Western blot of Fig. 5c.

Fig. S5f: full Western blot of Fig. 6b.

Fig. S5g: full Western blot of Fig. 6c.

Fig. S5h: full Western blot of Fig. 6d.

Fig. S5i: full Western blot of supplementary Fig. S3a.

a

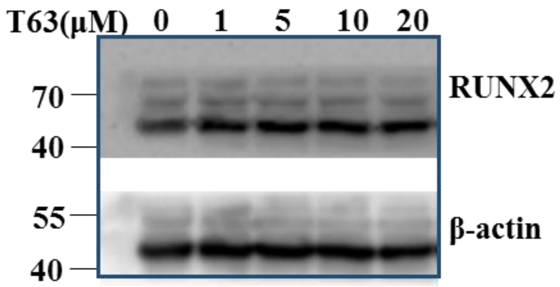

b

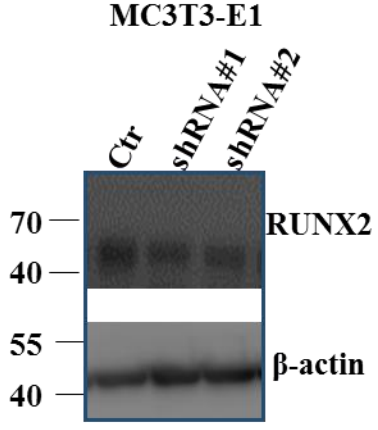

c

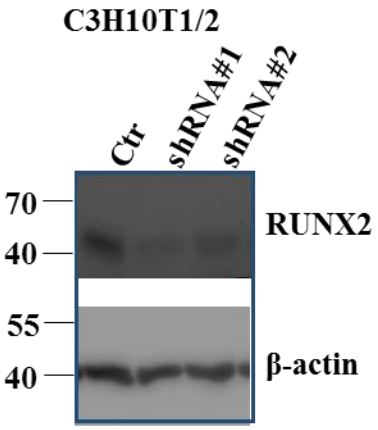

d

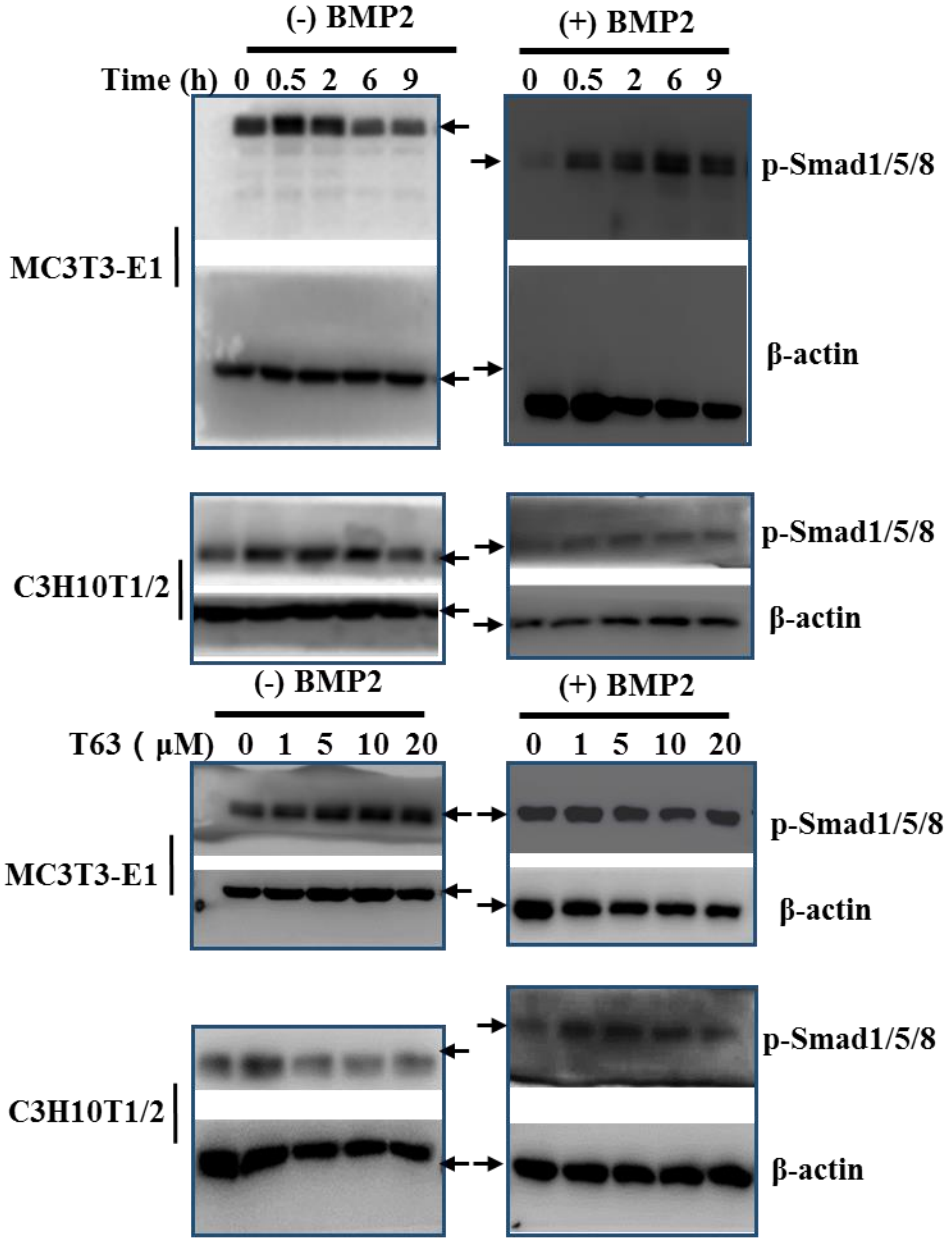

(-) BMP2
(+) BMP2
Time (h) 0 0.5 2 6 9
0 0.5 2 6 9
MC3T3-E1
p-Smad1/5/8
β-actin
C3H10T1/2
p-Smad1/5/8
β-actin
(-) BMP2
(+) BMP2
T63 (μM) 0 1 5 10 20
0 1 5 10 20
MC3T3-E1
p-Smad1/5/8
β-actin
C3H10T1/2
p-Smad1/5/8
β-actin

e

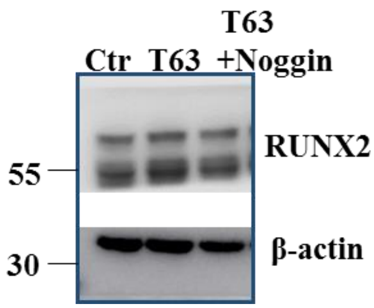

Ctr
T63
T63 +Noggin
RUNX2
β-actin
55
30

f

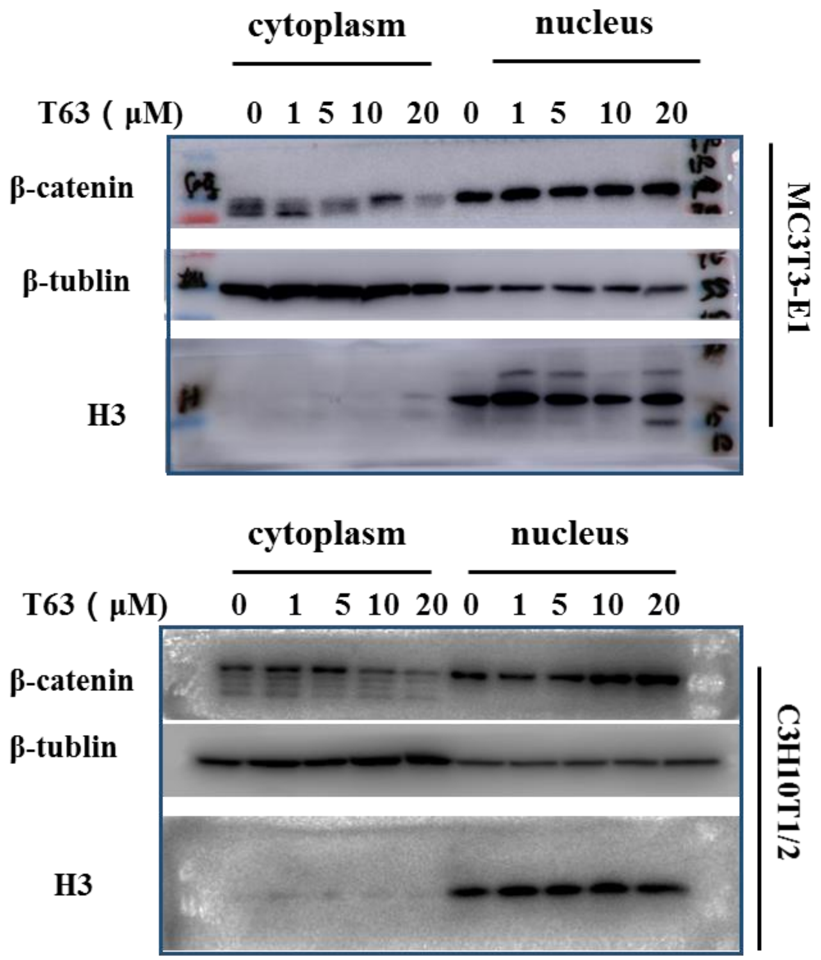
cytoplasm
nucleus
T63 (μM) 0 1 5 10 20 0 1 5 10 20
β-catenin
β-tublin
H3
MC3T3-E1
cytoplasm
nucleus
T63 (μM) 0 1 5 10 20 0 1 5 10 20
β-catenin
β-tublin
H3
C3H10T1/2

g

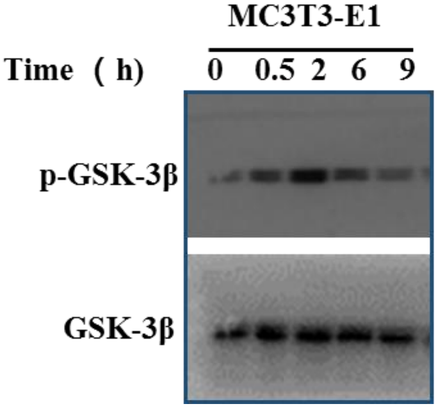
MC3T3-E1
Time (h) 0 0.5 2 6 9
p-GSK-3β
GSK-3β

h

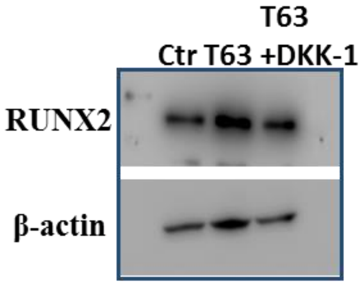
T63
Ctr T63 +DKK-1
RUNX2
β-actin

i

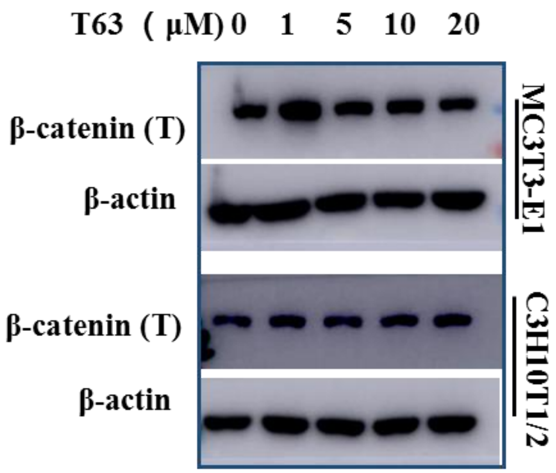
T63 (μM) 0 1 5 10 20
β-catenin (T)
β-actin
β-catenin (T)
β-actin
MC3T3-E1
C3H10T1/2

S6. Uncropped gel for Figures

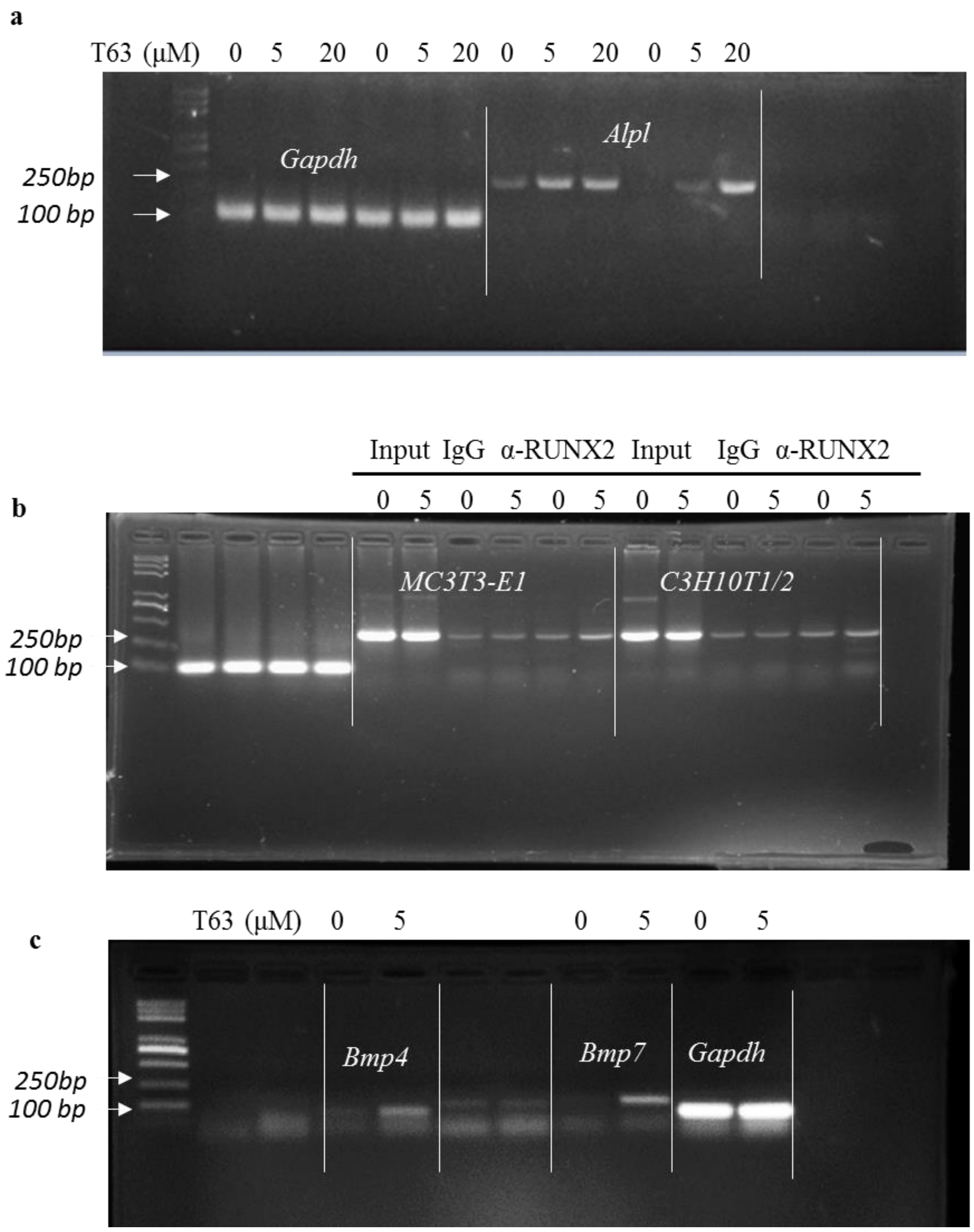

Fig. S6a: full gel of Fig.2b.

Fig. S6b: full gel of Fig. 4b.

Fig. S6c: full gel of supplementary Fig. S1b.
